# A recombinant fusion protein containing a spider toxin specific for the insect voltage-gated sodium ion channel shows oral toxicity towards insects of different orders

**DOI:** 10.1016/j.ibmb.2014.01.007

**Published:** 2014-04

**Authors:** Sheng Yang, Prashant Pyati, Elaine Fitches, John A. Gatehouse

**Affiliations:** a School of Biological and Biomedical Sciences, Durham University, South Road, Durham DH1 3LE, UK; b Fera (Food and Environment Research Agency), Sand Hutton, York, UK

**Keywords:** Neurotoxin, Insecticide, Recombinant protein expression system, Protein transport, Crop protection, Biotechnology

## Abstract

Recombinant fusion protein technology allows specific insecticidal protein and peptide toxins to display activity in orally-delivered biopesticides. The spider venom peptide δ-amaurobitoxin-PI1a, which targets insect voltage-gated sodium channels, was fused to the “carrier” snowdrop lectin (GNA) to confer oral toxicity.

The toxin itself (PI1a) and an amaurobitoxin/GNA fusion protein (PI1a/GNA) were produced using the yeast *Pichia pastoris* as expression host. Although both proteins caused mortality when injected into cabbage moth (*Mamestra brassicae*) larvae, the PI1a/GNA fusion was approximately 6 times as effective as recombinant PI1a on a molar basis. PI1a alone was not orally active against cabbage moth larvae, but a single 30 μg dose of the PI1a/GNA fusion protein caused 100% larval mortality within 6 days when fed to 3rd instar larvae, and caused significant reductions in survival, growth and feeding in 4th – 6th instar larvae. Transport of fusion protein from gut contents to the haemolymph of cabbage moth larvae, and binding to the nerve chord, was shown by Western blotting. The PI1a/GNA fusion protein also caused mortality when delivered orally to dipteran (*Musca domestica*; housefly) and hemipteran (*Acyrthosiphon pisum*; pea aphid) insects, making it a promising candidate for development as a biopesticide.

## Highlights

• The spider toxin Pl1a, specific for the insect sodium channel, was expressed alone and as a fusion with snowdrop lectin (GNA).
• Both recombinant toxin and fusion protein were insecticidal to housefly adults and cabbage moth larvae when injected.
• Only the fusion protein was orally toxic to housefly adults and cabbage moth larvae.
• The fusion protein showed enhanced oral toxicity towards aphids compared to its components.
• The Pl1a/GNA fusion protein has promise for development as a biopesticide.

## Graphical abstract

**Figure:**
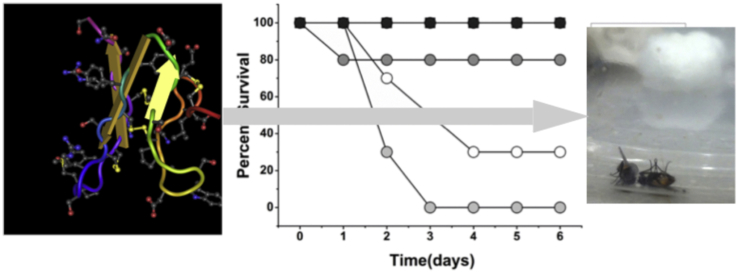

## 1 Introduction

Synthetic pesticides have been widely used for crop protection against herbivorous insects in intensive agricultural production, and are a necessary input to achieve high yields and consequent food security. However, there are widely held concerns over the indiscriminate use of pesticides in general, and insecticides in particular, including the development of resistance in target pests, detrimental effects in non-pest and beneficial insects, contamination of watercourses and the poisoning of higher animals. Therefore, many of the older, broad-spectrum insecticidal compounds have been, or are likely to be, withdrawn (Denholm and Rowland, 1992; Casida and Quistad, 1998; Desneux et al., 2007). Protein-based biopesticides fulfil many of the criteria required for more environmentally compatible approaches to pest control, since they combine efficacy with specificity, and are biodegradable in the environment. Besides naturally occurring protein biopesticides such as *Bacillus thuringiensis* toxins, biotechnological methods can be used to produce recombinant proteins with insecticidal activity. These include insecticidal fusion proteins containing a toxic peptide or protein fused to a “carrier”, where the carrier confers oral activity on a toxin that must normally be injected into the insect to reach its site of action, by directing transport of the fusion across the insect gut (Fitches et al., 2004).

Venoms isolated from a range of arachnids have been shown to contain proteins which are biologically active toxins when injected into potential prey. Most are small proteins, in the range 30–70 amino acid residues (variously referred to as peptides or proteins), that principally target neuronal ion channels, and to a lesser extent neuronal receptors and presynaptic membrane proteins, to cause paralysis of the prey (Rash and Hodgson, 2002). As a result of evolutionary selection, some toxins combine a high toxicity for insects with no effects on members of other taxons (Vassilevski et al., 2009). The potency and selective mode of action of spider neurotoxins would make them ideal candidates for use in environmentally compatible pest management technologies, if a suitable delivery system could be devised (Whetstone and Hammock, 2007). In general, these toxins are not effective as oral or contact insecticides, and no system which requires injection could possibly be feasible in the field.

Biopesticides used for crop protection against insect pests generally function via oral delivery, with the toxin proteins present in, or sprayed on plant tissues susceptible to damage. The use of a “carrier” in recombinant fusion proteins leads to transport of toxin proteins from the gut contents across the insect gut epithelium to the central nervous system where the toxin is active, resulting in dramatically enhanced oral insecticidal activity (Fitches et al., 2002). The mannose-specific lectin from snowdrop (*Galanthus nivalis* agglutinin: GNA) has proved successful as a carrier. It is resistant to proteolytic activity in the insect gut, and can bind to gut epithelial glycoproteins, leading to transport into the haemolymph following ingestion. For example, a toxin protein from the spider *Segestria florentina* was delivered to the haemolymph of lepidopteran larvae after oral delivery by fusing to GNA, causing decreased survival and growth in insects fed on diet containing the fusion protein (Fitches et al., 2004). Fusion proteins are required to possess good stability, so they cannot be degraded in the environment or digested by gut enzymes of pests, and high toxicity, with activity towards pests comparable to the toxin proteins themselves.

δ-Amaurobitoxins, or δ-palutoxins, from the spider *Pireneitega luctuosus* (Araneae: Amaurobiidae; previously referred to as *Paracoelotes luctuosus*) are a family of four similar 36–37 residue peptides, designated Pl1a-d (Corzo et al., 2000). They contain 8 cysteine residues, which are disulphide-linked to form a cysteine knot motif. The δ-amaurobitoxins are effective insecticides, with an estimated LD50 values of 0.95–4.48 μg/100 mg when injected into lepidopteran larvae (oriental leafworm moth; *Spodoptera litura*). They show insect-specific toxicity, with no effects observed for Pl1a, Pl1c and Pl1d after intravenal injection in mice. The amaurobitoxins specifically target insect sodium channels, and their solution structure has elucidated the nature of the interaction (Corzo et al., 2005; Ferrat et al., 2005). The toxin Pl1a was selected as the subject of this study as it combines the highest insecticidal activity with no observed toxicity towards higher animals, and thus would be suitable as a biopesticide.

The present paper reports the production, purification and biological activity of recombinant δ-amaurobitoxin PI1a, and the fusion protein PI1a/GNA comprised of PI1a linked to the N-terminus of GNA. It shows that fusion to GNA enhances the insecticidal activity of Pl1a. Not only was fusion protein more toxic than recombinant PI1a when injected into cabbage moth (*Mamestra brassicae*) larvae, but it also had effective oral toxicity when the toxin alone did not. The fusion protein was also orally toxic to insects of different orders, pea aphids (*Acyrthosiphon pisum*; Hemiptera) and housefly (*Musca domestica*; Diptera). The PI1a/GNA fusion protein has potential to be a useful biopesticide for crop protection in the future.

## 2 Materials and methods

### 2.1 Materials

Chemicals and reagents were of analytical grade and were supplied by Sigma or BDH Chemical Company otherwise unless stated. Restriction enzymes and other molecular biology reagents were supplied by Fermentas. A double stranded DNA incorporating a sequence encoding the mature PI1a toxin (P83256), with codon usage optimised for yeast, was designed by the authors, synthesised and supplied by ShineGene Molecular Biotech, Inc. (Shanghai 201109, China; http://www.synthesisgene.com/) in the vector pUC57. Other oligonucleotides were supplied by Sigma Chemical Co. Recombinant snowdrop lectin was produced by the authors by expression in *Pichia pastoris*, as described by Baumgartner et al. (2004).

*Mamestra brassicae* (cabbage moth) were maintained in a licenced growth facility. The cultures were subject to a 16 h light, 8 h dark cycle and maintained at 25 °C, 40% relative humidity on a standard lepidopteran diet as described before (Bown et al., 1997). *M. domestica* (housefly) larvae were maintained on a wheat flour diet under similar conditions; adults were given 10% sucrose solution *ad libitum*. *A. pisum* (pea aphid) was cultured on plants of *Vicia faba* (broad bean cv. Sutton Dwarf) under conditions of 12 h light, 12 h dark, 18 °C, 70% relative humidity.

### 2.2 Expression constructs

The Pl1a coding sequence was transferred from pUC57 to the yeast expression vector pGAPZαB (Invitrogen; www.invitrogen.com) by digestion with *Pst*I and *Xba*I, isolation of the coding sequence fragment by agarose gel electrophoresis, followed by ligation to pGAPZαB which had been restricted with the same enzymes. DNA fragments were separated by agarose gel electrophoresis prior to ligation, and were purified from excised gel slices using a QuiaQuick Gel Extraction Kit (Qiagen; www.qiagen.com) as described in the manufacturer's protocol. After ligation, the resulting recombinant plasmid was cloned using standard protocols by transformation of electro-competent cells of *E. coli* (Sambrook and Russell, 2001). Selected clones were checked for correct assembly of the construct by DNA sequencing.

To produce a construct encoding the Pl1a/GNA fusion protein, the mature PI1a coding sequence from a verified expression construct in pGAPZαB was excised by digestion with *Pst*I and *Not*I, and purified by agarose gel electrophoresis as described above. A pGAPZαB plasmid containing the fusion protein construct Hv1a/GNA (Fitches et al., 2012) was digested with *Pst*I and *Not*I to remove the Hv1a coding sequence, and purified by agarose gel electrophoresis. The Hv1a coding sequence was then replaced by Pl1a by ligating the purified fragments, and cloning the resulting recombinant plasmid. Selected clones containing the expression vector encoding the PI1a/GNA fusion protein were verified by DNA sequencing. All DNA sequencing was carried out using Applied Biosystems ABI Prism 3730 automated DNA sequencers by DBS Genomics, School of Biological and Biomedical Sciences, Durham University, UK.

### 2.3 Expression of PI1a and PI1A/GNA fusion proteins in yeast

pGAPZα plasmids containing the PI1a and PI1a/GNA expression constructs were amplified in *E. coli*, purified and linearized with *Bln*I. Linearised plasmids were transformed into *Pichia pastoris* strain SMD1168H (Invitrogen) using the EasyComp Transformation kit (Invitrogen) as described in the manufacturer's protocol. Transformed yeast clones were plated and selected on YPG agar plates (1% yeast extract (w/v), 2% peptone (w/v), 4% glycerol (v/v), 1.5% agar (w/v)) containing zeocin (100 μg/ml). Selected clones (at least 10 for each construct) were checked for expression of recombinant proteins by analysis of culture supernatant from small-scale shake flask cultures grown for 2–3 days in YPG–zeocin media at 30 °C. Samples of supernatant were separated by SDS-polyacrylamide gel electrophoresis; gels were blotted onto nitrocellulose and probed with anti-(His)_6_ primary antibodies (Bio-Rad) or anti-GNA primary antibodies, followed by washing, probing with HRP-conjugated secondary antibodies (Bio-Rad), and detection of bound antibodies by ECL, as described previously (Fitches and Gatehouse, 1998).

Selected clones of *P. pastoris* containing the integrated PI1a and PI1a/GNA constructs were grown in a 7.5 L BioFlo 110 bench-top fermenter (New Brunswick Scientific). For fermentation, two 100 ml YPG cultures of *P. pastoris* containing toxin or fusion genes were grown for 2–3 days at 30 °C with shaking, prior to being used to inoculate 2.5 L of sterile minimal media supplemented with PTM1 salts. Cultivation at 30 °C, 30% dissolved oxygen; pH 4.5 with continuous agitation was continued with a glycerol feed (5–10 ml/h) over a period of 4 days (Fitches et al., 2004). Culture supernatant was separated from cells by centrifugation (20 min at 5000 g), filtered through GF/D and GF/F glass fibre membranes (Whatman) and adjusted to 0.02 M sodium phosphate buffer, 0.4 M sodium chloride, pH 7.4 by adding 4× concentrated stock.

Recombinant proteins were purified from clarified culture supernatant by nickel affinity chromatography on 5 ml HisTrap crude nickel columns (GE Healthcare) with a flow rate of 2 ml/min. After loading, the columns were washed with 0.02 M sodium phosphate buffer, 0.4 M sodium chloride pH 7.4 and the bound proteins were eluted with 0.2 M imidazole in the same buffer. Eluted proteins were checked for purity by SDS-PAGE, dialysed against deionised water using multiple changes to remove all small molecules, and freeze-dried.

### 2.4 Protein characterisation

Amounts of recombinant proteins were quantitatively estimated by comparison to known amounts of GNA standards run on SDS-PAGE gels, or by BCA analysis using a BCA™ Protein Assay Kit (Thermo Scientific). For N-terminal sequencing, proteins were separated by SDS-PAGE and blotted onto PVDF membrane. Excised bands were supplied for N-terminal sequencing to a commercial protein sequencing service (Shanghai Applied Protein Technology Co., Ltd, China). For further characterisation, recombinant PI1a and PI1a/GNA fusion protein were denatured by dissolving in 6 M urea and incubating at room temperature for 15 min prior to addition of SDS-sample buffer, and analysis by SDS-PAGE.

The presence of N-linked glycosylation on recombinant proteins was shown by treatment with PNGase F, using a deglycosylation kit from Biolabs Co., Ltd, UK as described in the manufacturer's protocol. 1 μl Glycoprotein Denaturing Buffer was added to 6 μg protein (dissolved in 5 μl 1× PBS), and the mixture was incubated 10 min at 100 °C. After cooling, 2 μl 10× G7 Reaction Buffer, 2 μl 10% NP40 and 1.5 μl PNGase F were added to a final volume of 20 μl, and the mixture was incubated at 37 °C for 1 h. The proteins were then analysed by SDS-PAGE. A reaction in which PNGase was omitted was used as a control.

### 2.5 Bioassays on cabbage moth larvae

Injection bioassays were carried out using 4–5th stadium *M. brassicae* larvae (approx.45–55 mg in weight) by injecting 5 μl of aqueous solution containing varying doses of PI1a and PI1A/GNA dissolved in 1× PBS (phosphate buffered saline; 0.15 M NaCl, 0.015 M sodium-phosphate buffer, pH 7.2). Controls were injected with 5 μl 1× PBS. For each dose, 30 larvae were injected and paralysis and mortality were scored at 12, 24, 36, 48, 60 and 72 h post injection including control. To estimate LD_50_ values, mortality at 48 h was used, to make results comparable to those of Corzo et al. (2000).

Droplet-feeding assays were conducted to assess the oral activity of PI1A/GNA towards *M. brassicae* third to sixth instar larvae. Larvae were fed once with a 2 μl droplet containing 20 or 30 μg of PI1A/GNA, 30 μg of PI1a, or 30 μg of GNA in 1× PBS and 10% sucrose. Smaller larvae had exposed repeatedly until they finished the 2 μl droplet. Control larvae were fed on droplets containing 1× PBS and 10% sucrose solution. Treated larvae were placed in ventilated plastic pots (250 ml) with standard artificial diet after consumption of the droplet. To encourage droplet consumption, larvae were starved for approx. 24 h prior to feeding. Larval weight and survival was recorded daily after droplet feeding. In experiments to determine effect on feeding, the artificial diet was weighed prior to introduction, and re-weighed on removal to determine the amount consumed; diet was replaced daily.

To study fusion protein uptake into insects, fifth instar larvae were droplet-fed with a sub-lethal dose (20 μg) of Pl1a/GNA fusion protein as described above, and then transferred back to standard rearing diet, to give a “feed-chase” experiment. Haemolymph samples were extracted from M. brassicae larvae at different intervals (2–72 h) after feeding as described previously (Fitches et al., 2012), and the extracted haemolymph was quantified for protein content (BCA™ Protein Assay Kit, Pierce; www.piercenet.com). Tissues (midgut, Malpighian tubules, fat body, nerve chord) were dissected from selected larvae, and extracted as described previously (Fitches et al., 2012). Western blotting of larval haemolymph and tissue samples was carried out using anti-GNA antibodies (1:3300 dilution) as described previously (Fitches et al., 2001). Similar methods were used to follow the fate of Pl1a/GNA fusion protein (20 μg doses) injected into the haemolymph of 5th instar *M. brassicae* larvae.

### 2.6 Bioassays on houseflies

Adults of *M. domestica* were injected with 1.0 μl of aqueous solution containing varying doses of PI1a and PI1a/GNA dissolved in 1× PBS, using a conventional Hamilton syringe with a fine needle. Survival was monitored over a 144 h period. In feeding assays, adult flies were allowed to feed from cotton pads which had been soaked in a solution containing varying concentrations of Pl1a/GNA in 60% sucrose; survival was monitored over a 120 h period in which flies were exposed continuously to the treatment.

### 2.7 Bioassays on pea aphids

The toxicity of proteins to *A. pisum* was determined by bioassay using a liquid artificial diet (Prosser and Douglas, 1992), using a parafilm sachet to deliver diet to insects. Proteins were dissolved in sterile diet at known concentrations. The standard assay used 1–2 day-old aphid nymphs, which had been conditioned by transfer to diet without added proteins prior to receiving the protein treatments, and continued the assay until the insects became mature. For experiments to investigate retention of proteins in aphids, PI1a/GNA, PI1a and GNA were labelled with fluorescein isothiocyanate (FITC) by mixing together equimolar concentrations of FITC (solution in dimethyl sulphoxide) and PI1a/GNA, PI1a and GNA (solutions in 1× PBS) (0.02 mg of FITC/mg of PI1a/GNA, 0.04 mg of FITC/mg of PI1a and 0.03 mg of FITC/mg of GNA, respectively). Aphids were fed on diets containing labelled proteins for 24 h, then transferred to control diet for a “chase” period of up to 48 h. Labelled proteins were detected by fluorescence microscopy of whole insects.

### 2.8 Statistical analysis

Survival data were analysed using Kaplan–Meier survival analysis, using Prism (v. 5) software. All other data analysis was carried out using Origin 8.5 graphing and data analysis software. ANOVA analysis (with Bonferroni–Dunn post-hoc tests) was carried out to determine any significant differences between treatments in the parameters measured.

## 3 Results

### 3.1 Expression and purification of recombinant PI1a and PI1a/GNA

**Fig. 1:**
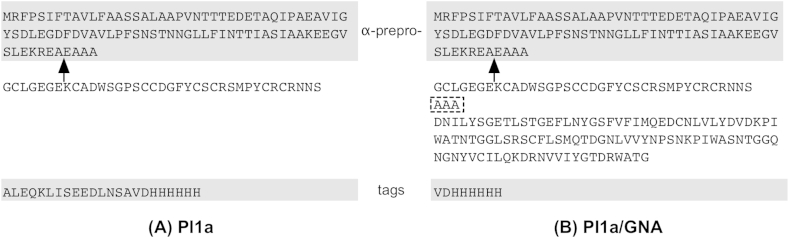
Sequences of predicted products from expression constructs for Pl1a toxin (A) and Pl1a/GNA fusion protein (B). Shaded regions indicate sequence provided by vector; the cleavage point for removal of the yeast α-factor prepro-sequence is indicated by an arrow. Dotted box in (B) indicates the “linker” sequence contributed by the nucleotides used to join the Pl1a and GNA coding sequences together.

Expression constructs for production of recombinant proteins in the methylotrophic yeast *Pichia pastoris* were based on the vector pGAPZα, which contains a constitutively expressed promoter and integrates into the yeast genome at the *GAPDH* locus, giving stable transformants. The expression construct for production of recombinant Pl1a contained a synthetic coding sequence corresponding to the published amino acid sequence for the toxin, arranged in-frame C-terminal to a sequence encoding the yeast α-factor prepro-sequence, and N-terminal to sequences encoding the myc epitope and (His)_6_ tag, supplied by the vector (Fig. 1A). The expression construct for production of recombinant PI1a/GNA fusion protein contained the same synthetic mature PI1a coding sequence fused to the N-terminus of a coding sequence corresponding to residues 1–105 of mature snowdrop lectin (GNA) via a 3 amino acid linker peptide; again, the fusion protein was arranged in-frame C-terminal to the α-factor prepro-sequence, and N-terminal to a sequence encoding the (His)_6_ tag, supplied by the vector (Fig. 1B). The constructs were assembled by restriction-ligation and were checked by DNA sequencing after cloning.

Verified clones of expression constructs were transformed into the protease-deficient *P. pastoris* strain SMD1168H, using antibiotic (zeocin) selection for transformants. Approx. 50 resistant colonies were obtained for each expression construct. Culture supernatant from selected clones grown in shake-flask cultures was analysed for production of recombinant proteins by Western blotting, to allow selection of clones producing the highest levels of PI1a and PI1a/GNA. Screening of large numbers of transformed yeast clones was not necessary, since most clones were expressing recombinant proteins, as judged by the presence of immunoreactive bands of the expected size on Western blots of culture supernatants.

For each construct, the best-expressing clone of those screened in small-scale cultures was selected for large-scale protein production by bench top fermentation. Culture supernatants were purified by nickel affinity chromatography, and eluted peaks were desalted by dialysis, and lyophilized. Yields of recombinant proteins were comparable to other fusion proteins prior to optimisation; Pl1a was produced at approx. 26 mg/L and PI1A/GNA at approx. 21 mg/L, as estimated by semi-quantitative SDS-PAGE.

**Fig. 2:**
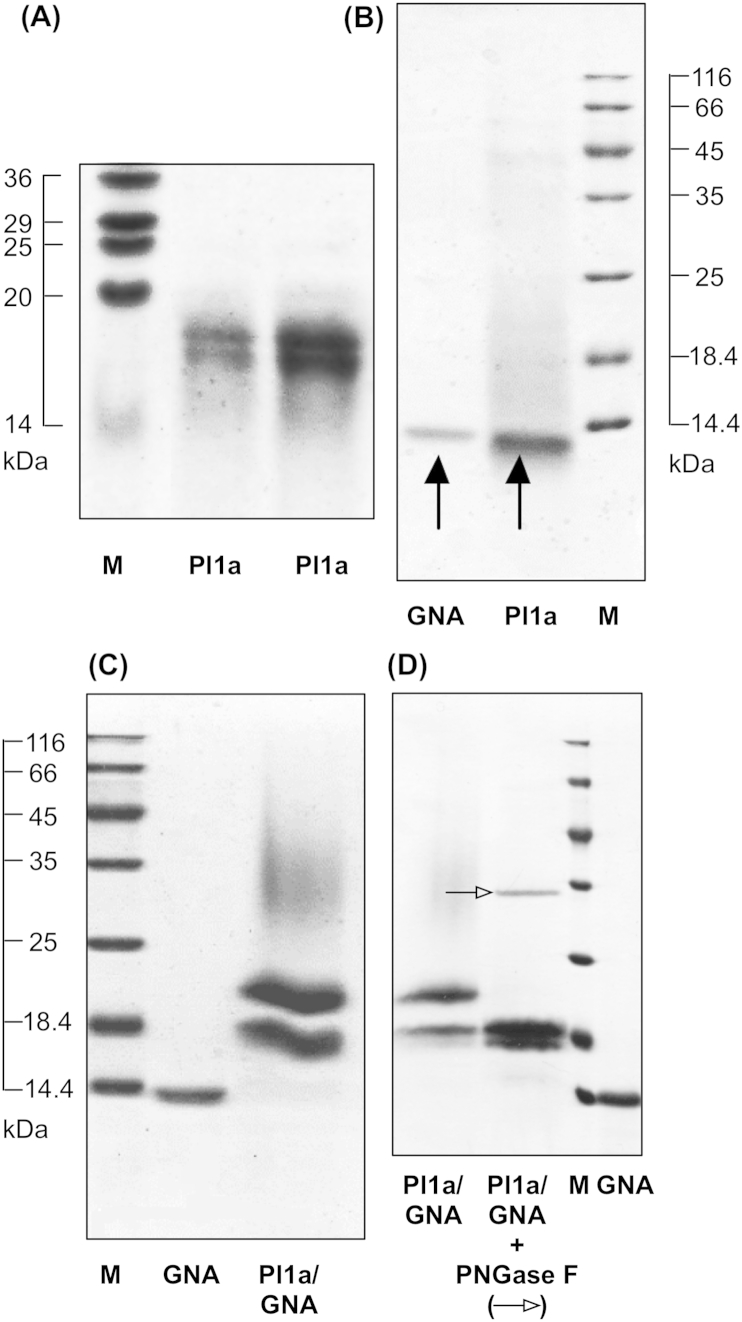
Characterisation of purified recombinant proteins by SDS-PAGE followed by Coomassie blue staining. (A) Pl1a toxin separated on “normal” SDS-PAGE; M indicates marker, loadings of Pl1a are 5 and 10 μg (B) Pl1a toxin (5 μg) separated on SDS-PAGE after denaturation by 6 M urea. (C) Pl1a/GNA fusion protein (10 μg). (D) Deglycosylation of Pl1a/GNA fusion protein using PNGase F (band indicated by open arrowhead).

Purified recombinant proteins were analysed by SDS-PAGE and western blot. The recombinant toxin Pl1a (Fig. 2A) ran as a closely spaced double band at an indicated mol. wt. of approx. 18 kDa on normal SDS-PAGE gels; both bands were immunoreactive with anti-(His)_6_ antibodies on Western blotting (not presented). The predicted mol. wt. of recombinant Pl1a, including the tag sequences is 7.07 kDa. The double band and incorrect mol. wt. of toxin was reproducible with different gels, samples, and use of reducing agents prior to electrophoresis, but was considered to be an artefact of the gel system, possibly as a result of poor binding of SDS to the polypeptide. When the same samples were treated with 6 M urea prior to electrophoresis, Pl1a gave a single band at an indicated mol. wt. of 14 kDa (Fig. 2B); the shift in mobility is indicative of gel artefacts, and the single band indicates homogeneity of the product. Further analysis on urea-containing gels gave single bands for Pl1a, with indicated mol. wts. of approx. 11 kDa without blocking cysteine residues, and approx. 9 kDa after treatment with iodoacetamide to block cysteine residues (data not presented); these results are diagnostic of incorrect mol. wts. under “normal” conditions due to residual secondary structure and interactions between cysteine residues prior to or during electrophoresis.

The Pl1a/GNA fusion protein (Fig. 2C) contained a closely spaced double band of an indicated size of 18 kDa, similar to the expected molecular weight for the fusion protein (17.3 kDa); pretreatment of samples with 6 M urea caused a slight shift in molecular weight to a lower value, and replacement of the double band by a single band, once again suggesting the double band was an artefact (data not presented). The N-terminal sequence of the single band was determined as E-A-A-A-G-, as expected for the fusion protein after removal of the yeast α-factor prepro-region during translation and secretion from *Pichia*. The fusion protein gave two further bands on gel when analysed by SDS-PAGE. It contained a small amount of a band at an indicated molecular weight similar to recombinant GNA (12.7 kDa), which was immunoreactive to anti-GNA antibodies, suggesting a small amount of cleavage of the fusion protein into its components was occurring during production and purification. The ratio of intact PI1a/GNA fusion protein to cleaved GNA was estimated as approx. 30:1 as judged by Coomassie blue staining on SDS-PAGE gels. The Pl1a/GNA fusion protein also contained a prominent band at an indicated mol. wt. of approx. 21 kDa, roughly equal in intensity to the band assumed to be Pl1a/GNA fusion protein. This was again immunoreactive with anti-GNA antibodies, and had an identical N-terminal sequence to the 18 kDa band. Treatment with the deglycosylating enzyme PNGase F, which cleaves carbohydrate side chains attached to Asn residues through N-glycosidic bonds, removed this band, while the intensity of the “correct” band for the Pl1a/GNA fusion protein increased as a result of the treatment (Fig. 2D). This result suggests that the extra band is due to “core” glycosylation of the fusion protein by *P. pastoris* during synthesis and secretion. GNA contains no potential N-glycosylation sites, but the Pl1a toxin sequence contains a potential N-glycosylation site (N-X-S/T) at Asn-35. Quantitation of the Pl1a/GNA fusion protein was based on the combined intensity of both the bands representing the glycosylated and non-glycosylated forms. Treatment with PNGase F also removed a “smear” of material of higher molecular weight on SDS-PAGE in Pl1a/GNA, which was assumed to represent hyper-glycosylated fusion protein.

### 3.2 Toxicity of proteins to cabbage moth larvae after injection into the haemolymph

**Fig. 3:**
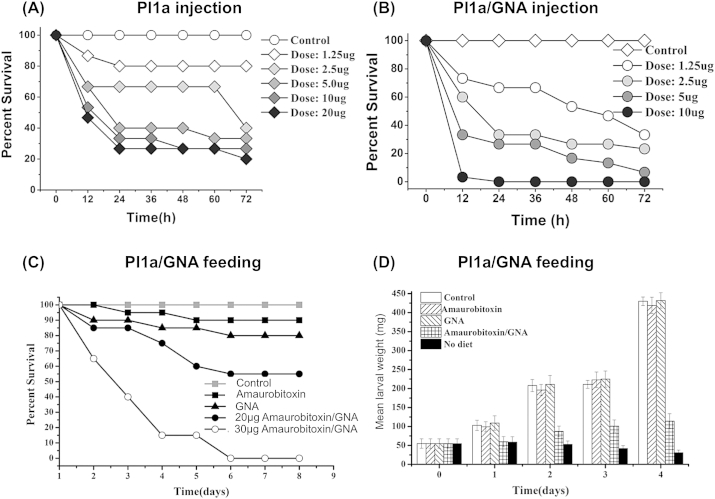
Bioassays of Pl1a and Pl1a/GNA against larvae of cabbage moth (*Mamestra brassicae*). (A) Survival of 5th instar larvae after injection of varying amounts of Pl1a toxin. (B) Survival of 5th instar larvae after injection of varying amounts of Pl1a/GNA fusion protein. (C) Survival of 3rd instar larvae after feeding a single dose of Pl1a (Amaurobitoxin; 30 μg), snowdrop lectin (GNA; 30 μg) or Pl1a/GNA fusion protein (Amaurobitoxin/GNA; dose as indicated). (D) Growth of 5th instar larvae after feeding a single dose of Pl1a (Amaurobitoxin; 30 μg), snowdrop lectin (GNA; 30 μg) or Pl1a/GNA fusion protein (Amaurobitoxin/GNA; 30 μg).

Newly eclosed 5th instar larvae (approx. 45–55 mg in weight; average weight 50 mg) of *M. brassicae* were injected with recombinant PI1a and PI1a/GNA fusion protein to assay biologically activity *in vivo*. Larvae injected with PI1a toxin all displayed flaccid paralysis within 1–2 h (little mobility and almost a complete absence of feeding). Most mortality was observed within the first 24 h of the assay (Fig. 3A). After a period of paralysis, some insects showed progressive recovery, and were able to recommence feeding. The effects of Pl1a were dose dependent, with mortality after 24 h ranging from 75% at 20 μg toxin/insect to 20% at 1.25 μg toxin/insect. Even at high doses of toxin, complete mortality after 72 h was not observed. From these assays, the estimated LD_50_ (48 h) for the recombinant Pl1a was 4.1 μg/insect, or 8.2 μg/100 mg insect, based on an average larval weight of 50 mg.

The Pl1a/GNA fusion protein also caused paralysis and mortality when injected into *M. brassicae* larvae, but was significantly more effective than toxin alone. When insects were injected with 1.25–10 μg fusion protein/insect (equivalent to 0.50–4.0 μg PI1a/insect, since the molecular weight of recombinant Pl1a is approx. 0.41 of that of the PI1a/GNA fusion protein), significant mortality was observed at all doses, and complete mortality at 24 h was observed at the highest dose (Fig. 3B). As observed for Pl1a, most mortality occurred within the first 24 h of the assay, and effects of PI1a/GNA fusion protein were dose dependent, ranging from 100% mortality at 10 μg fusion protein/insect to 33% mortality at 1.25 μg fusion protein/insect after 24 h. Mortality at this lowest dose of fusion protein increased to 67% after 72 h whereas mortality from injection of 1.25 μg toxin alone/insect did not change from 20% in the period 24–72 h. From these assays, the estimated LD_50_ (48 h) for the recombinant Pl1a/GNA fusion protein was 1.4 μg/insect, or 2.8 μg/100 mg insect, based on a mean larval weight of 50 mg. The LD_50_ estimated for fusion protein is equivalent to 0.57 μg of recombinant Pl1a toxin per insect, making the fusion protein approx. 7.5 times as active as the recombinant toxin. A similar ratio is obtained by using mortality figures at 72 h. Direct comparisons of mortality produced by identical doses of toxin and fusion protein show that the treatments are different from each other, and from control, at *p* < 0.0001 (ANOVA). In all these assays, no mortality of control injected insects was observed over 72 h.

### 3.3 Toxicity of proteins to cabbage moth larvae after oral delivery

Newly hatched third instar larvae of *M. brassicae* could consume up to 2 μl droplets of phosphate buffered saline (PBS) containing 10% w/v sucrose if starved for 24 h prior to the experiment. This method was used to deliver recombinant proteins to assay their oral toxicity, by dissolving the protein in the PBS/sucrose solution. Two doses of PI1a/GNA fusion protein (20 μg and 30 μg per droplet) and one dose each of Pl1a (30 μg) and GNA (30 μg) were delivered as experimental treatments. Control larvae were fed PBS/sucrose. Results are shown in Fig. 3C.

Effects on mortality caused by the different treatments were observed over the first 6 days of the assay, with no further effects up to day 8; control survival was 100% over this period. All protein treatments caused reduced survival, but the Pl1a toxin effect was not significant (survival analysis, log rank test), causing only 10% mortality. The effect of GNA, which caused 20% mortality, was just significant (*p* = 0.037). In contrast, both doses of fusion protein caused highly significant effects on survival (*p* < 0.01). A single 30 μg dose of the Pl1a/GNA fusion protein led to complete larval mortality after 6 days, with most mortality occurring in the first 4 days after exposure; the 20 μg dose of fusion protein caused 45% mortality. Insects exposed to fusion protein showed partial paralysis, and became lethargic and unresponsive.

Toxic effects were also observed when Pl1a/GNA fusion protein was fed to larger larvae. Newly eclosed fifth instar larvae fed a single dose of 30 μg of PI1a/GNA fusion protein showed 35% mortality over 4 days, whereas control larvae or larvae fed 30 μg doses of PI1a or GNA exhibited 100% survival (significantly different; *p* < 0.0001). Surviving insects which had been fed the fusion protein showed strongly retarded growth, increasing their weight only two-fold over 4 days, whereas control insects increased their weight 8-fold (Fig. 3D). Insects, which had been fed Pl1a or GNA showed no difference in weight gain to the control. The difference in mean larval weight values between fusion-exposed and control, GNA and PI1a treatments was highly significant (*P* < 0:0001; ANOVA). The effect of Pl1a/GNA fusion protein was not the same as starvation, since insects fed no diet showed a weight loss (final weight: initial weight = 0.57) over this period. Instead, oral administration of the fusion protein caused reduced feeding after administration. Insects were transferred back to standard rearing diet, and consumption of diet was measured by decrease in wet weight. The consumption of diet by insects was correlated with their weight gain; larvae fed diet containing fusion protein consumed approx. 10% of the diet consumed by controls over 5 days, whereas consumption by larvae fed Pl1a or GNA did not differ significantly from controls (result not presented). The reduced diet consumption is consistent with the observation that insects consuming fusion protein became lethargic and unresponsive, even after transfer back to rearing diet.

### 3.4 Detection of ingested PI1a/GNA in cabbage moth larval tissues after oral delivery

**Fig. 4:**
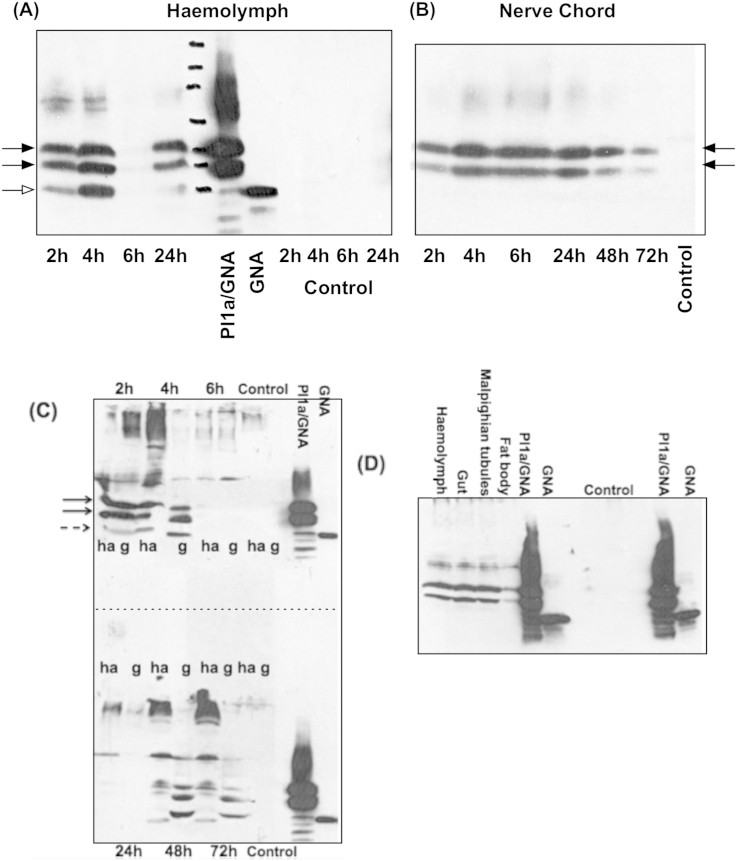
Transport of Pl1a/GNA fusion protein from gut contents to haemolymph, Malpighian tubules, fat body and nerve chord in larvae of cabbage moth (*Mamestra brassicae*). Larvae were injected or fed a single dose of Pl1a/GNA fusion protein, and tissues were sampled at the indicated time after feeding. The presence of Pl1a/GNA fusion protein was visualised by SDS-PAGE analysis of extracted proteins, followed by western blotting using anti-GNA antibodies. (A) Haemolymph from treated and control insects after feeding Pl1a/GNA. (B) Nerve chords from treated and control insects after feeding Pl1a/GNA. (C) Gut and haemolymph 2 h, 4 h, 6 h, 20 h, 24 h and 48 h, respectively, after feeding Pl1a/GNA; ha, haemolymph; g, gut. (D) Various tissues 4 h after injection of Pl1a/GNA to hemolymph.

To establish that the PI1a/GNA fusion protein was capable of transporting across the gut in *M. brassicae* larvae, haemolymph was extracted from insects fed on diets containing fusion protein and was analysed for the presence of fusion protein by western blotting, using anti-GNA antibodies (Fig. 4A). Insects were starved, given a single 20 μg dose of Pl1a/GNA in liquid diet, and then returned to normal rearing diet, so the experiment is essentially a “pulse-chase”. The blot confirmed that intact PI1a/GNA fusion protein was present in treated insects after 2 h, whereas control insects showed no immunoreactive material. The western blot showed evidence for partial proteolysis of the Pl1a/GNA fusion protein, with increased levels of a band corresponding in size to GNA being visible on the blots in comparison to purified fusion protein; the sample taken 4 h after feeding the protein shows a “GNA” band comparable in intensity to the fusion protein bands, whereas in the purified protein the “GNA” band is present only at very low intensity compared to the fusion protein bands. The time course of accumulation of fusion protein in the haemolymph gave an unexpected result in that levels of Pl1a/GNA in the haemolymph increased from the 2 h after feeding sample to 4 h, but the haemolymph sample taken 6 h after feeding contained only very small amounts of Pl1a/GNA compared to the 4 h sample; this result was reproducible over different feeding experiments. Samples taken at later times (24–72 h) showed fusion protein present in haemolymph at higher levels than at 6 h after feeding.

One destination of Pl1a/GNA fusion protein delivered to the haemolymph was the central nervous system, the site of action of the toxin. This was shown by dissection of nerve chords from insects after feeding, and analysis by Western blotting (Fig. 4B). Proteins extracted from nerve chords showed immunoreactivity with anti-GNA antibodies, at a level that increased from 2 to 4 h after feeding, and then remained similar for up to 24 h. The immunoreactive bands indicated a higher level of intact fusion protein than the product of proteolysis, GNA. Levels of fusion protein in the nerve chord then declined from 24 h to 72 h after feeding. This accumulation of GNA-based neurotoxic fusion proteins on the nerve chord of insects has been observed previously by direct visualisation using labelled proteins (Fitches et al., 2012). Further examination of tissues from insects fed a “pulse” of Pl1a/GNA fusion protein confirmed the disappearance of immunoreactive bands from gut and haemolymph 6 h after feeding, and a reappearance of the fusion protein and GNA after 24 h, first in the haemolymph at 24 h after feeding and then in the gut at 48 h and 72 h after feeding (Fig. 4C). These results suggest that the Pl1a/GNA fusion protein initially binds to nervous tissue, but is subsequently released back into the haemolymph, and subsequently reassociates with gut tissue. In a confirmatory experiment, Pl1a/GNA fusion protein was injected into the haemolymph of *M. brassicae* larvae at sub-lethal levels, and was detected in different tissues after 4 h (Fig. 4D). Pl1a/GNA was found associated with gut tissue and Malpighian tubules; a small of amount of protein was also present in fat body. No evidence of proteolytic cleavage of this material to GNA was observed, confirming that haemolymph contains low levels of proteolytic activity, in contrast to high levels of protease in the gut, where cleavage of the fusion protein was observed after 4 h when Pl1a/GNA was delivered orally (Fig. 4A).

### 3.5 Effects of Pl1a and Pl1a/GNA fusion protein on housefly

**Fig. 5:**
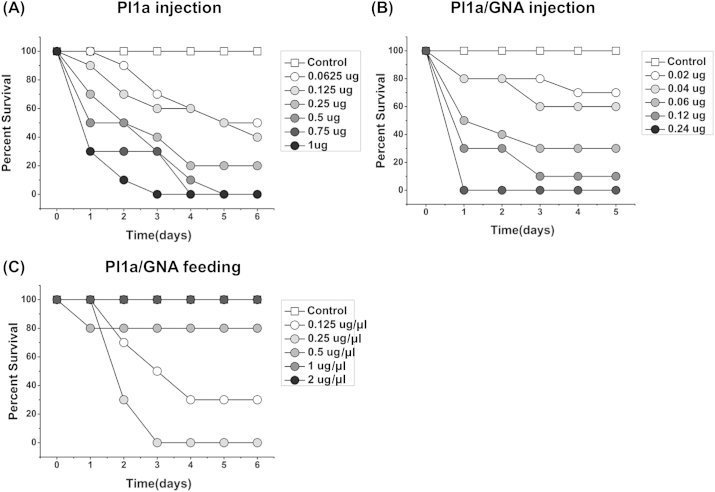
Bioassays of Pl1a and Pl1a/GNA against adults of housefly (*Musca domestica*). (A) Survival of adult flies after injection of varying amounts of Pl1a toxin. (B) Survival of adult flies after injection of varying amounts of Pl1a/GNA fusion protein. (C) Survival of adult flies allowed to feed *ad libitum* on solutions containing Pl1a/GNA fusion protein (Amaurobitoxin/GNA; concentration as indicated).

Bioassays using housefly were carried out on adult insects, which could be injected using basic equipment without causing high levels of mortality. These assays showed that both the recombinant Pl1a toxin and the Pl1a/GNA fusion protein caused paralysis and mortality when injected. Typical results are shown in Fig. 5A and B. Mortality was dose dependent, with most insect deaths taking place in the first 72 h after injection. A dose of 1.0 μg of recombinant Pl1a caused 100% mortality in 72 h, and doses ≥0.5 μg caused 100% mortality in 144 h The data gave an estimated LD_50_ (72 h) of 0.18 μg Pl1a per insect, or approx. 1.8 μg Pl1a per 100 mg insect, based on an average adult weight of approx. 10 mg. The Pl1a/GNA fusion protein was significantly more effective than the recombinant toxin, with more rapid mortality at lower doses; at a dose of 0.24 μg per insect, 100% mortality was observed after 24 h. The estimated LD_50_ (72 h) for the fusion protein was 0.045 μg per insect, or approx. 0.45 μg fusion protein per 100 mg insect; this is equivalent to 0.18 μg of Pl1a, making the fusion protein approx. 10 times as effective, on a mole-for-mole basis, as the recombinant toxin. The Pl1a/GNA fusion protein was also an effective toxin when fed to adult *M. domestica* (Fig. 5C); a 0.25 μg/μl solution caused 100% mortality in 72 h, whereas 0.125 μg/μl solution caused 70% mortality. Flies were completely paralysed approx. 2 h after feeding, and most paralysed insects subsequently died. Higher concentrations of the fusion protein caused lower mortality over 6 days, as the insects would not feed, or fed only very little; the chambers were moist enough to allow insects to survive without feeding.

Attempts to inject larvae of *M. domestica* also showed that both the toxin Pl1a and the Pl1a/GNA fusion protein were effective toxins, but control survival in these assays was erratic due to damage from the injection. Larvae could not be induced to feed on material containing recombinant proteins.

### 3.6 Effects of oral delivery of Pl1a/GNA fusion protein on pea aphids

**Fig. 6:**
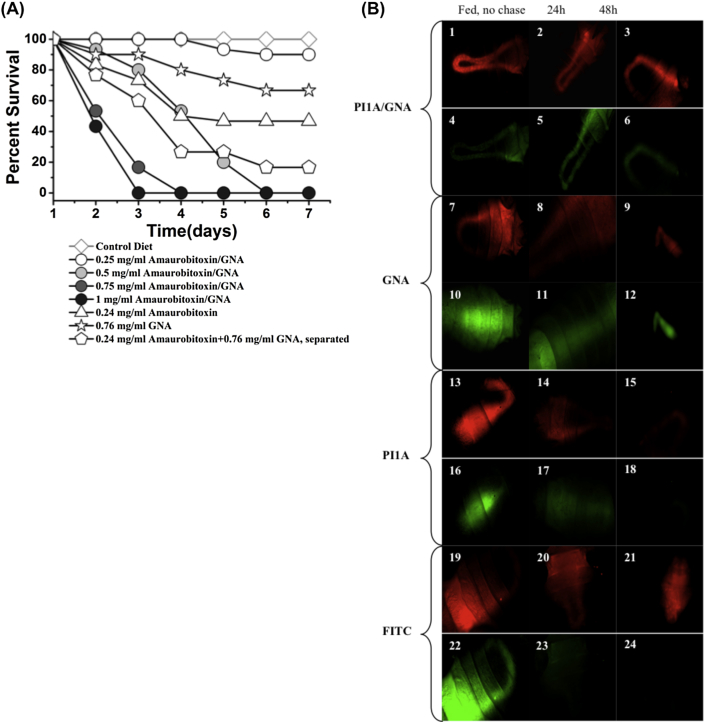
Bioassays of Pl1a and Pl1a/GNA against nymphs of pea aphid (*Acyrthosiphon pisum*). (A) Survival of aphids on diets containing Pl1a (Amaurobitoxin), snowdrop lectin (GNA) or Pl1a/GNA fusion protein (Amaurobitoxin/GNA) at concentrations as indicated. (B) Feed-chase experiment to show binding of proteins to aphid gut. Diets containing recombinant proteins labelled with FITC were fed to aphids for 24 h. Subsequently the label was “chased” with control diet for times as indicated. Red fluorescence indicates the aphid gut, green fluorescence indicates labelled proteins. (For interpretation of the references to colour in this figure legend, the reader is referred to the web version of this article.)

Purified recombinant PI1a, Pl1a/GNA fusion protein and recombinant GNA were fed to *A. pisum* nymphs by incorporation into artificial diet at a range of concentrations (Fig. 6A). Survival and growth of the insects were monitored. Aphids feeding on 1.0 mg/ml Pl1a/GNA fusion protein showed 100% mortality in 3 days of feeding, which was significantly different to negative controls, whereas aphids feeding on diet containing 0.24 mg/ml Pl1a or 0.76 mg/ml GNA showed only 53.3% or 33.3% mortality in 7 days of feeding compared with 1.0 mg/ml Pl1a/GNA. Moreover, feeding a mixture of Pl1a (0.24 mg/ml) and GNA (0.76 mg/ml), which was equivalent to 1 mg/ml Pl1a/GNA fusion protein in the content, showed 83.3% mortality in 7 days of feeding whereas 1 mg/ml Pl1a/GNA caused 100% mortality in 3 days. The fusion protein survival curve was significantly different to controls and other treatments (*p* < 0.001). The treatments also decreased in aphid growth by approx. 60%, which was significant compared to controls, but differences between treatments were not significant (data not presented).

Feeding Pl1a/GNA at different concentrations from 0.25 mg/ml to 1 mg/ml showed a dose dependent effect on *A. pisum* survival (data not presented). After 7 days of feeding, 1.0 mg/ml Pl1a/GNA caused 100% mortality whereas the lowest concentration of Pl1a/GNA, 0.25 mg/ml, produced approx. 10% mortality. From 0.5 mg/ml to 1 mg/ml, all survival curves for Pl1a/GNA were significantly different to negative controls, and aphid growth was significantly reduced. However, the size of 0.25 mg/ml Pl1a/GNA-fed aphids was not significantly different to control aphids, suggesting that the aphids were capable of overcoming the growth retardation effects of 0.25 mg/ml Pl1a/GNA.

To demonstrate binding of proteins to the aphid gut surface, recombinant Pl1a, GNA and Pl1a/GNA fusion protein were labelled by conjugation with fluorescein, and fed in diet to aphids at a sub-lethal concentration (0.8 mg/ml Pl1a, 1 mg/ml GNA and 0.64 mg/ml Pl1a/GNA) for 24 h. The label was then ‘‘chased’’ by allowing aphids to feed on control diet for 24 h and 48 h. Labelled proteins were detected in whole insects by fluorescence microscopy, and were readily detectable in insects with no chase after feeding. Results are presented in Fig. 6B. Fluorescein, used as a negative control, (Fig. 6B, panel 24), was eliminated completely from the aphid gut after 48 h chase, whereas fluorescein-labelled GNA, used as a positive control, was still present in the gut after 48 h chase (Fig. 6B, panel 12). As expected, the labelled Pl1a/GNA fusion protein also persisted in the midgut region of aphids, and was detectable even after 48 h chase (Fig. 6B, panel 6). Surprisingly, labelled recombinant Pl1a could also bind to the gut (Fig. 6B, panels 16–17), and was detectable after 24 h chase, although the level of binding after 48 h chase decreased to undetectable, (Fig. 6B panel 18), in contrast to labelled GNA and Pl1a/GNA, which were detectable after 48 h chase. These results showed that although recombinant Pl1a, GNA, and Pl1a/GNA could all bind to the aphid gut, recombinant Pl1a was most readily removed, suggesting weaker binding.

## 4 Discussion

The δ-amaurobitoxin PI1a was selected as a possible component for biopesticidal fusion proteins for reasons described earlier (insecticidal activity and insect-specificity), but also because it is effective against a different target than previous insecticidal neurotoxins used in lectin-based fusion proteins; Sfl1, from the spider *Segestria florentina* (Fitches et al., 2004) has an unknown target, ButaIT from the scorpion *Mesobuthus tamulus* (Trung et al., 2006) is assumed to target chloride channels, and Hv1a, from the spider *Hadronyche versuta* (Fitches et al., 2012) targets calcium channels. As a toxin which targets the insect sodium channel (Corzo et al., 2005), Pl1a therefore represents a novel type of insecticidal component. The insect sodium channel is a major target for conventional pesticides, such as pyrethroids, and inactivation leads to rapid paralysis and death; exploitation of this target in the insect is thus based on established practice.

Pl1a represents a distinct type of sodium channel inactivating toxin (Ferrat et al., 2005). Although most spider toxins just slow NaCh inactivation in a fashion similar to that of receptor site 3 modifiers, δ-amaurobitoxins are similar to scorpion β-toxins in binding with high affinity to the topologically distinct receptor site 4, which involves domain II in insect and mammalian NaChs (Cestele et al., 1998). δ-amaurobitoxins and scorpion β-toxins show some similarity in their bioactive surfaces and ability to compete for an identical receptor (site 4) on voltage-gated NaChs, though they have developed from different ancestors. The δ-amaurobitoxins like PI1a recognize insect voltage-gated sodium channels by multiple sequence features, including a β-sheet secondary structure, loops I, IV of the toxin and the specific dipolar moment orientation (Ferrat et al., 2005). The roles of different amino acid residues in determining binding and toxicity have been investigated by alanine scanning mutagenesis; Asp-19 may be causal in toxicity, since substitution of this residue by Ala affected toxicity to lepidopteran larvae, but not binding to the sodium channel (Corzo et al., 2005). These results can be exploited to manipulate the toxin component of a fusion protein if necessary to modify activity or specificity. Data for the insecticidal activity of Pl1a (Arachnoserver) suggests that it shows a higher LD_50_ on a mole/g basis than than that reported for Hv1a, the toxin component of the atracotoxin/GNA fusion protein described by Fitches et al. (2012).

The yeast *Pichia pastoris* was selected as expression host for production of recombinant PI1a and Pl1a/GNA fusion proteins on the basis of previous work showing that small proteins containing multiple disulphide bonds can be produced in active form in this organism (Cereghino and Cregg, 2000). Efficient secretion of expressed proteins into the culture medium, directed by the yeast α-factor prepro-sequence incorporated into the expression vector pGAPZα, is an additional advantage in that purification of the recombinant protein is simplified by having relatively few contaminating *Pichia* proteins present in the culture medium, and not having to lyse cells to obtain the product. Proteolysis of fusion proteins produced in *P. pastoris* during secretion, or in the culture medium, or during purification, has been a significant problem with previous toxin-GNA fusions (Fitches et al., 2004), resulting in the final product containing significant amounts (up to 50%) of GNA without attached toxin. However, the Pl1a/GNA fusion protein is relatively resistant to proteolysis, and the purified product contains only small amounts of free GNA.

*P. pastoris* has an efficient N-glycosylation system for proteins which pass through the ER, although in most cases core glycosylation with a branched oligomannose structure is only elaborated by addition of extra mannose residues (Bretthauer and Castellino, 1999). Both the recombinant Pl1a toxin and the Pl1a/GNA fusion protein contain an N-glycosylation site, corresponding to the sequence –NNS– at the C-terminus of the mature toxin. However, only the fusion protein shows evidence of glycosylation at this site. Utilisation of an N-glycosylation site requires the amino acid residues to be accessible to the glycosylating enzyme(s), and not all sites are used. The difference in glycosylation properties of the recombinant toxin and fusion protein is evidence for differences in folding and accessibility in this region of the toxin.

Both the recombinant toxin alone and the fusion protein, show insecticidal activity on injection into lepidopteran and dipteran insects, with the expected symptoms of paralysis and mortality. However, in both sets of assays, the fusion protein has an activity at least 6-fold higher on a molar basis than the recombinant toxin. There is some evidence from injection bioassays to suggest that the recombinant toxin has lower insecticidal activity than expected. The LD_50_ for recombinant toxin alone observed in the injection bioassays against *M. brassicae* larvae, 4.1 μg/insect, or 12 nmoles/g insect is approx. 5-fold higher than the quoted literature value for purified and synthetic Pl1a toxins of 2.35 nmoles/g insect for larvae of *S. litura* [LD_50_ (48 h) = 9.5 μg/g insect; Corzo et al., 2000]. In contrast, the LD_50_ for the recombinant fusion protein is lower than this literature value for purified toxin when expressed on a molar basis; 1.4 μg/insect for Pl1a/GNA is equivalent to 1.6 nmoles/g insect. If it is assumed that larvae of the two lepidopteran species have similar susceptibility to the Pl1a, then these data would suggest that the toxin in the Pl1a/GNA fusion protein has the expected biological activity, whereas the recombinant toxin alone does not. Two possibilities can be advanced to explain this observation. First, fusion to GNA could improve toxin folding during production as a recombinant protein, leading to a product with more biological activity. Secondly, the carbohydrate-binding activity of GNA enables it to act as an anchor to bind toxin to nerve tissue and increase its local concentration, leading to a higher effective dose. The evidence from western blotting showing high levels of fusion protein associated with nerve chord tissue supports this hypothesis. The results presented here, in agreement with previous data (Fitches et al., 2012), show that fusion to GNA can enhance recombinant toxin biological activity.

The droplet feeding assays provide clear evidence of the oral toxicity of the Pl1a/GNA fusion protein towards lepidopteran and dipteran insects. In the assays with *M. brassicae* larvae no significant toxicity of the toxin alone was observed, and only marginal effects from the GNA carrier, in agreement with previous assays in which GNA was fed to larvae of tomato moth, *Lacanobia oleracea* (Fitches et al., 2001). Only the fusion protein was tested against *M. domestica* adults, but previous results have shown that GNA alone has only limited toxicity at high doses (Fitches et al., 2009). The resistance to proteolysis shown by the Pl1a/GNA fusion protein, observed during production and purification, is likely to be a factor in its oral toxicity; a high proportion of the GNA transported across the gut will be fused to the toxin, resulting in efficient transport of toxin into the haemolymph (free toxin does not transport, since orally delivered toxin is ineffective). However, some cleavage of fusion protein to release GNA does occur in the larval gut, since significant levels of free GNA are subsequently present in the haemolymph, and fusion protein injected into the haemolymph is stable. High levels of proteolytic activity are present in the larval gut of M. *brassicae* (Chougule et al., 2008).

The western blotting experiments show transport of intact fusion protein into the haemolymph, and accumulation on nervous tissue; after feeding a single dose, fusion protein initially accumulates in the haemolymph, and then clears after 6 h. The subsequent reappearance of fusion protein in haemolymph after 24 h is most likely to be due to release from nervous tissue that is being degraded, as a result of partial or complete inactivation due to the toxin. Although the initial transport of fusion protein is from gut to haemolymph, interestingly, retrograde transport of fusion protein from haemolymph to gut can also occur, suggesting that transport across the gut is a passive, rather than an active process.

Whereas the Pl1a/GNA fusion protein shows effective oral toxicity in the lepidopteran and dipteran insects tested while its component proteins either have no toxicity, or very limited toxicity, the situation is less clear cut in aphids. The fusion protein was a more effective toxin than either of its components, or a mixture of its components, but both components of the fusion showed significant oral toxicity. For GNA, this is in agreement with previous reports of oral toxicity to aphids and other hemipteran insects. The oral toxicity of the Pl1a toxin itself is more surprising, and the mechanism through which the toxin is able to access sites of action when fed to aphids remains obscure. Further experiments will be necessary to show whether the binding of toxin to the gut surface in aphids leads to transport to the haemolymph (as is the case for GNA) or whether the toxin remains in the gut contents. In this example, fusion to GNA enhances the oral toxicity of Pl1a rather than conferring novel oral toxicity.

The amaurobitoxin-lectin fusion protein described in this paper is a promising candidate for development as a biopesticide with activity against lepidopteran and dipteran pests; it has an approx. 10-fold lower LD_50_ towards *M. brassicae* larvae by injection than the Hv1a/GNA fusion protein described by Fitches et al. (2012), and caused mortality after droplet feeding a single dose to 5th instar larvae of *M. brassicae*, whereas a greater dose of Hv1a/GNA fusion protein only caused growth retardation. It is also approx. 8-fold more active towards *M domestica* adults than the ButaIT/GNA scorpion toxin fusion protein described by Fitches et al. (2009), where a 1.0 μg/μl solution caused only 75% mortality after 72 h, in contrast to 70% mortality produced by a 0.125 μg/μl solution of Pl1a/GNA. Further trials of insecticidal activity and selectivity will be necessary to ensure that the fusion protein could be used safely in agricultural applications.
